# Cognitive Syndromes and C9orf72 Mutation Are Not Related to Cerebellar Degeneration in Amyotrophic Lateral Sclerosis

**DOI:** 10.3389/fnins.2019.00440

**Published:** 2019-05-10

**Authors:** Monica Consonni, Eleonora Dalla Bella, Anna Nigri, Chiara Pinardi, Greta Demichelis, Luca Porcu, Cinzia Gellera, Viviana Pensato, Stefano F. Cappa, Maria Grazia Bruzzone, Giuseppe Lauria, Stefania Ferraro

**Affiliations:** ^1^3rd Neurology Unit and Motor Neuron Diseases Centre, Fondazione IRCCS Istituto Neurologico Carlo Besta, Milan, Italy; ^2^Neuroradiology Department, Fondazione IRCCS Istituto Neurologico “Carlo Besta,” Milan, Italy; ^3^Laboratory of Methodology for Clinical Research, Oncology Department, IRCCS Istituto di Ricerche Farmacologiche Mario Negri, Milan, Italy; ^4^Unit of Genetics of Neurodegenerative and Metabolic Diseases, Motor Neuron Diseases Centre, Fondazione IRCCS Istituto Neurologico Carlo Besta, Milan, Italy; ^5^Institute for Advanced Study-IUSS Pavia, Pavia, Italy; ^6^IRCCS Centro San Giovanni di Dio Fatebenefratelli, Brescia, Italy; ^7^Department of Biomedical and Clinical Sciences “Luigi Sacco”, University of Milan, Milan, Italy

**Keywords:** cerebellum, amyotrophic lateral sclerosis, cortical thickness, cortical volume, cognitive impairment

## Abstract

**Objective:**

The notion that cerebellar pathology may contribute to cognitive impairment in ALS, especially in patients with C9orf72 repeated expansion, has been inconsistently reported. This study aimed exploring the relationship between cerebellar involvement, cognitive impairment and C9orf72 repeated expansion of patients with ALS.

**Methods:**

Quantitative *in vivo* assessment of cerebellar lobules has been investigated in 66 non-demented patients with ALS and 28 healthy controls (HCs). Pathologic C9orf72 repeated expansion was found in 13 patients. Mild cognitive and/or behavioral impairment was diagnosed in 22 C9orf72 negative ALS patients. Measures of cortical volume (CV) and cortical thickness (CT) of cerebellar lobules of all participants were used for Principal Component Analysis (PCA) to identify clusters of lobular measures highly correlated with each other. PCA outcomes were used for between group comparisons and correlation analyses with neuropsychological and clinical features.

**Results:**

Disease severity measured with ALS functional rating scale and index of disease progression rate significantly correlated with CV reduction of the second PCA cluster loading CV measures of anterior lobules. In all patients, cognitive impairment, measured with verbal fluency, was related to CV reduction of the third cluster comprising posterior lobules. No specific cortical thinning or volume reduction of cerebellar clustering patterns could be detected in ALS subgroups.

**Conclusion:**

Our data show that specific patterns of subregional cerebellar involvement are associated with physical disability or cognitive impairment in ALS, in line with the topographic organization of the cerebellum. However, there was no specific correlation between cerebellar degeneration and cognitive syndromes or C9orf72 mutations.

## Introduction

The human cerebellum has been long recognized as playing an essential role in motor control and coordination and it has also become increasingly aligned with cognitive and affective processing (Schmahmann and Sherman, 1998; Buckner, 2013). Functional neuroimaging and connectivity studies in healthy humans suggest a topographic organization in the cerebellum: sensorimotor functions are represented in anterior lobules I–V and inferior lobule VIII, while cognitive processing is supported by posterior lobules (Stoodley and Schmahmann, 2009). Cerebellar abnormalities have been found not only in primary cerebellar injury or degeneration, but also in many psychiatric and neurological diseases without ataxia, including Amyotrophic Lateral Sclerosis (ALS) (Gellersen et al., 2017; Carass et al., 2018).

ALS is a fatal multisystemic neurodegenerative disorder mainly characterized by progressive loss of upper and lower motor neurons with cognitive and/or behavioral disturbance in at least half of cases. ALS-associated cognitive syndrome includes impairment of executive function, often with perseveration, distractibility or inattention, language deficit and social cognition impairment (Strong et al., 2017). Personality change might also occur with apathy, blunting of affect or disinhibited and inappropriate behavior (Strong et al., 2017). In a subset (15%) of patients these cognitive and neuropsychiatric changes are severe enough to fulfill criteria for the behavioral variant of frontotemporal dementia (bvFTD) (Phukan et al., 2012). ALS patients with the C9orf72 hexanucleotide repeat expansion (C9+ALS) had significantly more co-morbid bvFTD features than those without (Byrne et al., 2012). Cognitive and neuropsychiatric features of ALS have largely been attributed to changes in the frontotemporal and insular cortex (Tsujimoto et al., 2011; Agosta et al., 2016; Westeneng et al., 2016; Consonni et al., 2018a,b; Christidi et al., 2018a), although increasing evidence suggest hippocampus and subcortical region degeneration also plays a role (Bede et al., 2013b; Machts et al., 2015; Westeneng et al., 2016), especially in C9+ALS patients (Bede et al., 2013a; Agosta et al., 2017; Floeter et al., 2018; Floeter and Gendron, 2018).

Evidence of cerebellar involvement in ALS is less commonly reported (Prell and Grosskreutz, 2013). Structural changes of the cerebellum in ALS have been described as regional white matter changes (Bede et al., 2015) and decrease gray matter volume (Thivard et al., 2007; Gellersen et al., 2017; Christidi et al., 2018a,b). Cerebellar changes in ALS and FTD have been specifically linked to the C9orf72 repeat expansion (Mackenzie et al., 2013), raising the question whether cerebellar degeneration is an important feature of C9+ALS.

In order to explore the relationship between cerebellar involvement, cognitive impairment and C9orf72 repeated expansion of patients with ALS, quantitative *in vivo* assessment of cerebellar lobules has been investigated in a large sample of non-demented ALS patients stratified on the basis of cognitive and/or behavioral involvement and C9orf72 mutation.

## Materials and Methods

### Participants

Sixty-six non-demented patients diagnosed with probable or definite ALS, according to El Escorial revised criteria (Brooks et al., 2000) and 28 healthy control (HC) volunteers were included in the study. Exclusion criteria were comorbid frontotemporal dementia (Rascovsky et al., 2011), Alzheimer’s disease (NIA-AA), evidence of another neurologic condition affecting cognition (e.g., head trauma, hydrocephalus, and vascular disease), drug or alcohol abuse, mental retardation, primary psychiatric disorders, and other severe medical conditions. The review board of the Fondazione IRCCS Istituto Neurologico Carlo Besta of Milan approved the study. Each subject was enrolled after giving written informed consent.

### ALS Subgroups

Patients were assigned to subgroups on the basis of the genetic screening for C9orf72 mutations and on the basis of the presence of cognitive (ALSci) and/or behavioral (ALSbi) impairment as defined by recent guidelines (Strong et al., 2017). Accordingly, a cognitive screening protocol was administer to all participants (Consonni et al., 2016; Table 1). Our cohort was composed of 13 patients with a pathological C9orf72 expansion (C9+ALS) and 53 non-affected C9orf72 patients (C9-ALS). Among C9-ALS patients, 31 patients had normal cognitive profile (C9-ALScn) and 22 had ALSci and/or ALSbi (C9-ALSimp).

**Table 1:** Demographic, clinical and neuropsychological data (mean ± standard deviation).

	C9-ALScn *N* = 31	C9-ALSimp *N* = 22	C9+ALS *N* = 13	HC *N* = 28	Group comparisons
**Demographic data**					
Age (yrs.)	58.22 ± 9.4	60.81 ± 11.7	57.76 ± 8.5	57.28 ± 10.1	n.s.
Education (yrs.)	11.29 ± 3.8	11.45 ± 4.1	8.53 ± 3.2	11.82 ± 3.7	C9+ALS < HC^∗^
Male / Female	16 / 15	11 / 11	4 / 9	12 / 16	n.s.
Handedness (EHI)	21.60 ± 3.9	20.00 ± 6.13	21.33 ± 6.1	22.04 ± 1.8	n.s.
**Clinical data**					
ALSFRS-R	39.22 ± 5.6	37.95 ± 7.6	39.00 ± 6.09	–	n.s.
Disease duration (mos.)	16.81 ± 12.0	18.77 ± 13.5	13.15 ± 7.3	–	n.s.
Progression rate index	0.65 ± 0.4	0.63 ± 0.4	0.91 ± 0.8	–	n.s.
Bulbar onset (yes / no)	3 / 28	7 / 15	3 / 10	–	n.s.
**Neuropsychological data**					
ALSci / ALSbi / ALScbi	0 / 0 / 0	17 / 1 / 4	8 / 2 / 0	–	C9+ALS > C9–ALS^∗^
Phonemic fluency index	4.83 ± 1.9	6.90 ± 4.16	8.25 ± 7.9	4.17 ± 2.0	C9-ALSimp > HC^∗^
Object naming (% correct)	95.72 ± 3.8	86.34 ± 10.2	89.09 ± 13.1	94.76 ± 4.9	C9-ALSimp < HC^∗∗∗^ C9-ALSimp < C9-ALScn^∗∗∗^
Emotion attribution (SET)	4.89 ± 1.2	3.38 ± 1.56	3.36 ± 2.0	4.89 ± 1.1	C9-ALSimp < HC^∗∗^ C9-ALSimp < C9-ALScn^∗∗^
Stroop effect	19.02 ± 8.1	31.31 ± 24.1	25.08 ± 9.3	20.25 ± 7.9	C9-ALSimp < C9-ALScn^∗^
Delayed recall RAVLT	10.00 ± 2.5	7.62 ± 2.7	7.72 ± 3.28	10.36 ± 2.6	C9-ALSimp < HC^∗∗^ C9-ALSimp < C9-ALScn^∗∗^
FBI tot	1.96 ± 2.5	4.00 ± 6.7	2.03 ± 3.4	–	n.s.


*ALSbi, ALS patients fulfilling Strong criteria for behavioral impairment; ALSci, ALS patients fulfilling Strong criteria for cognitive impairment; ALScbi, ALS patients fulfilling criteria for ALSci and ALSbi; ALSFRS-R, Revised ALS Functional Rating Scale; FBI, Frontal Behavioral Inventory; RAVLT, Rey Auditory Verbal Learning Test; SET, Story-based Empathy Task; and ns, not significant difference (*p* > 0.05). ^∗∗∗^*p* < 0.001; ^∗∗^*p* < 0.005; ^∗^*p* < 0.05.*

### Magnetic Resonance Imaging

All patients and healthy participants underwent a magnetic resonance imaging (MRI) session within a week of neuropsychological assessment, on a Philips Achieva 3.0 T. The MRI protocol included a 3D T1-weighted sequence (FFE, 240 sagittal slices, TR = 9.9 ms, TE = 4.6 ms, matrix 240 × 240, voxel size = 1 × 1 × 1 mm3, and flip angle = 8). In order to have a quantitative evaluation of cerebellum lobules representing the current state of the art, we used the CERES automated cerebellum parcellation algorithm (Romero et al., 2017). With this tool, the MRI images are denoised, corrected for inhomogeneity, rigid-body registered to Montreal Neurological Institute (MNI) template, cropped around the cerebellum area, and normalized to the MNI cropped cerebellum atlas. An automatic multi-atlas patch-based segmentation was then applied to obtain cerebellar cortical thickness (CT) and volume (CV), expressed in percentage of total intracranial volume, for each lobule (Manjón and Coupe, 2016).

#### Cerebellar Cluster Identification

CT and CV measures over twelve cerebellar lobules were obtained. Principal Component Analysis (PCA) was then applied as an unbiased method to reduce the number of variables for subsequent between-group and correlation analyses. PCA with oblimin rotation was used to identify the clustering pattern (of highly correlated variables) of cerebellar CT and CV measures of all subjects. For this purpose, for each CT and CV measure, *z*-scores were calculated by subtracting the mean CT and CV of HC subjects from the patient’s CT and CV, and then dividing the difference by the HC group standard deviation. All variables were normally distributed and had Kaiser-Meyer-Olkin (KMO) values higher than 0.5. Clusters with eigenvalues over the Kaiser criterion of one were retained. Cluster scores were calculated with the Anderson-Rubin method and used for between group and correlation analyses (Field, 2013).

### Statistics

The Kolmogorov–Smirnov test was used to test the normality of the distribution of demographic, clinical, neuropsychological and neuroimaging data. Fisher Exact test, Kruskal–Wallis and Mann–Whitney *U* tests were used to test between group differences (i.e., C9-ALSimp, C9-ALScn, C9+ALS, and HC). The variability of cerebellar cluster loadings of groups was analyzed by ANCOVA, with age as covariates. Simple contrasts were then used to test specific group comparisons. Partial correlation analyses (correcting for age and education) were used to explore the relationship of cerebellar cluster scores with neuropsychological and clinical profiles in all patients as a whole group. Significant correlations were then bias corrected and accelerated bootstrap 95% confidence intervals (CIs) were computed with 1000 bootstrap equally sized samples obtained by randomly resampling replacement from the original data. Partial correlations with *p* < 0.05 and CIs not crossing “0” were reported. IBM SPSS Statistics (version 21) was used to perform analyses.

## Results

### Clinical, Demographic and Neuropsychological Data

Descriptive statistics are summarized in Table 1. C9-ALScn, C9-ALSimp, C9+ALS, and HC groups did not differ in to age, handedness, gender, but education (*H* = 9.019, *p* = 0.029). The C9+ALS group had a slightly lower education level than the HC group (*U* = 93.0, *p* = 0.012). Disease duration, functional disability assessed with the ALSFRS-R, progression and type of onset were similar across ALS subgroups. HC and C9-ALScn groups had similar neuropsychological performances. The occurrence of cognitive and/or behavioral impairment is higher for C9+ALS patients than C9-ALS patients (77% vs. 41%; *p* = 0.031). The C9-ALSimp group compared with HC and C9-ALScn groups had lower performances in all cognitive domains (Table 1).

### Cerebellar Clustering Patterns

**CTz.** The KMO measure tested the sample adequacy of 12 cerebellar lobular CTz values for PCA (KMO = 0.708). The rotation converged on 23 iterations. Four clusters had eigenvalues over Kaiser criterion of one and in combination explained 78.41% of the variance (Table 2). Cluster 1 included CTz values of lobule VIIIA, VIIIB, and IX; Cluster 2 included CTz values of Crus II and VIIB, Cluster 3 loadings were CTz values of lobule IV, V, VI, and Crus I; cluster 4 included lobule I&II and III and X.

**Table 2:** Pattern matrix of PCA results for cerebellar lobar values of Cortical Thickness (CT) of 94 subjects [66 patients with ALS and 24 healthy controls (HCs)].

				Rotated cluster loadings	
	Cluster 1	Cluster 2	Cluster 3	Cluster 4	KMO value
CTz Lobule IX	**0.882**	0.100	-0.165	0.181	0.752
CTz Lobule VIIIB	**0.854**	0.210	-0.246	0.398	0.746
CTz Lobule VIIIA	**0.704**	0.672	-0.120	0.220	0.725
CTz Lobule VIIB	0.255	**0.894**	0.004	-0.049	0.595
CTz Lobule CRUSII	0.053	**0.840**	-0.472	0.003	0.645
CTz Lobule VI	0.113	0.112	**-0.858**	0.144	0.822
CTz Lobule CRUS I	0.050	0.404	**-0.830**	0.048	0.693
CTz Lobule V	0.509	-0.160	**-0.767**	0.373	0.763
CTz Lobule IV	0.472	-0.184	**-0.745**	0.490	0.728
CTz Lobule I&II	0.221	-0.083	-0.114	**0.882**	0.685
CTz Lobule III	0.130	-0.195	-0.213	**0.861**	0.612
CTz Lobule X	0.247	0.138	-0.110	**0.454**	0.700
Eigenvalues	3.929	2.295	1.757	1.122	
% of variance	32.74	19.13	14.64	9.35	


*The loading values in bold indicate the elements that contribute the greatest variability to PCA clusters. ALScn, ALS patients with a cognitive and behavioral normal profile; ALSimp, ALS patients fulfilling Strong criteria for behavioral and/or cognitive impairment; C9+ALS, ALS patients with C9orf72 repeat expansions; and HC, Healthy Controls.*

**CVz.** The KMO measure tested the sample adequacy of 12 cerebellar lobular CV for PCA (KMO = 0.671). The rotation converged on 10 iterations. Four clusters had eigenvalues over Kaiser criterion of one and in combination explained 62.13% of the variance (Table 3). Cluster 1 included CVz of lobule VIIIB, IX, and X; Cluster 2 included CVz of anterior lobules (I&II, III, IV, and V); Cluster 3 loadings were CVz values of CrusII, VIIB and VIIIA; cluster 4 included CVz of lobule VI and Crus I.

**Table 3:** Pattern matrix of PCA results for cerebellar lobar values of Cortical Volumes (CV) of 94 subjects (66 patients with ALS and 24 HCs).

	Rotated cluster loadings				
	Cluster 1	Cluster 2	Cluster 3	Cluster 4	KMO value
CVz Lobule IX	**0.826**	-0.079	0.283	0.155	0.661
CVz Lobule VIIIB	**0.727**	0.391	0.153	0.108	0.690
CVz Lobule X	**0.651**	-0.054	0.324	0.494	0.779
CVz Lobule III	-0.035	**0.788**	0.264	0.040	0.655
CVz Lobule V	0.201	**0.684**	0.126	0.506	0.685
CVz Lobule I&II	0.057	**0.602**	0.002	-0.014	0.528
CVz Lobule IV	0.487	**0.498**	0.119	0.364	0.702
CVz Lobule CRUSII	0.298	0.066	**0.831**	-0.016	0.609
CVz Lobule VIIB	-0.092	0.130	**0.773**	0.284	0.668
CVz Lobule VIIIA	0.351	0.119	**0.692**	0.115	0.702
CVz Lobule VI	0.006	0.255	0.174	**0.809**	0.601
CVz Lobule CRUS I	0.339	-0.095	0.070	**0.697**	0.677
Eigenvalues	3.226	1.653	1.408	1.166	
% of variance	26.88	13.77	11.73	9.71	


*The loading values in bold indicate the elements that contribute the greatest variability to PCA clusters. ALScn, ALS patients with a cognitive and behavioral normal profile; ALSimp, ALS patients fulfilling Strong criteria for behavioral and/or cognitive impairment; C9+ALS, ALS patients with C9orf72 repeat expansions; and HC, Healthy Controls.*

We did not find group differences in cluster scores of CTz and CVz measures (Table 4). Correlation analyses revealed that only CVz scores of the second and third cluster were related to functional disability measured with the ALSFRS-R in all patients (*r* = 0.283, *p* = 0.024, CIs: 0.043–0.468; *r* = 0.281, *p* = 0.026, CIs: 0.053–0.467). Cluster 2 CVz scores were also related to the progression rate (*r* = -0.248, *p* = 0.050, CIs: -0.087 – -0.403). Cluster 4 CVz scores were inversely related to the phonemic fluency index (*r* = -0.335, *p* = 0.010, CIs: -0.157 – -0.535).

**Table 4:** Clustering pattern of cerebellar lobules (mean cluster score ± standard deviation).

	ALScn *N* = 31	ALSimp *N* = 22	C9+ALS *N* = 13	HC *N* = 28	Group comparisons
**Cortical thickness – cluster scores**					
1. Lobule VIIIA, VIIIB, IX	-0.278 ± 0.96	0.362 ± 0.75	-0.253 ± 1.27	0.141 ± 0.99	n.s.
2. Lobule Crus II, VIIB	-0.004 ± 1.14	0.076 ± 0.82	-0.531 ± 1.16	0.191 ± 0.83	n.s.
3. Lobule IV, V, VI, Crus I	-0.272 ± 0.89	-0.063 ± 0.80	0.463 ± 1.43	0.136 ± 0.95	n.s.
4. Lobule I&II, III, X	-0.122 ± 1.07	0.353 ± 0.88	0.125 ± 0.99	-0.200 ± 0.96	n.s.
**Cortical volumes – cluster scores**					
1. Lobule VIIIB, IX, X	0.038 ± 0.86	-0.191 ± 0.94	0.263 ± 1.44	-0.014 ± 0.95	n.s.
2. Lobule I&II, III, IV, V	0.064 ± 0.97	-0.086 ± 1.29	-0.014 ± 1.11	0.003 ± 0.72	n.s.
3. Lobule Crus II, VIIB, VIIIA	-0.042 ± 0.96	-0.323 ± 1.03	0.004 ± 0.99	0.299 ± 0.98	n.s.
4. Lobule VI, Crus I	0.201 ± 0.92	-0.306 ± 0.80	0.006 ± 0.87	0.014 ± 1.23	n.s.


*n.s., not significant differences (*p* > 0.05).*

## Discussion

We tested the relationship between cerebellar degeneration, cognitive syndromes and C9orf72 mutation in ALS patients. The notion that cerebellum contributes to non-motor disorders, such as impairments in language, attention and social cognition is well recognized (Buckner, 2013). Consistently, we performed a quantitative *in vivo* assessment of cerebellar lobular CT and CV in a large sample of non-demented ALS patients stratified on the basis of cognitive and/or behavioral impairment and C9orf72 repeat expansion. To this aim, we used an unbiased reduction method, the PCA, which allows projecting data into a lower-dimensional space thus reducing the type I error and identifying clusters of highly correlated variables with each other (Field, 2013).

We identified 4 clusters of regional cerebellar CT and demonstrated their scores were similar across ALS subgroups and HC individuals. Moreover, the lack of significant correlations between cerebellar CT values, neuropsychological performances and clinical features in patients suggests that the cerebellar cortical thinning has a poor sensibility to detect cognitive impairment in ALS. Cognitive and neuropsychiatric features of ALS seem to be associated instead with thinning in frontotemporal and insular cortex (Schuster et al., 2014; Agosta et al., 2016; Consonni et al., 2018a,b).

By means of lobar CV measures, we identified four clustering patterns. The clustering of the cerebellum respected lobular anatomical boundaries and functional organization. Cluster 1 included posterior regions (VIIIB-IX, X), cluster 2 the sensory-motor anterior regions (I-V), cluster 3 (CrusII-VIIIA), and 4 (VI-CrusI) included mainly cognitive posterior lobules. Contrary to our expectation, no significant group differences in cluster scores were found, but, as explorative analysis, a weak but significant correlation between CV measures of the fourth cluster and phonemic fluency indexes of ALS patients. Verbal fluency is a widely recognized marker of dysexecutive impairment in ALS (Strong et al., 2017). Specifically, poor verbal fluency was related to reduced CV of lobule VI and CrusI. This is consistent with previous studies documenting that lobules VI and Crus I are involved in executive functions, such as working memory, planning, organizing, and strategy formation (Stoodley and Schmahmann, 2009). According to a meta-analysis of voxel-based morphometry in cerebellum (Gellersen et al., 2017), the largest cluster of gray matter reduction in ALS spanned parts of the vermis and neighboring regions in left lobule VI, Crus I, and Crus II. This suggests that cerebellar involvement in ALS may be related to progressive cognitive impairment. However, as in other studies (Schönecker et al., 2018), we did not find differences in cerebellar volume within ALS cognitive subgroups, albeit specific patterns of cerebellum atrophy in ALS-FTD patients have been previously reported (Tan et al., 2014; Omer et al., 2017; Christidi et al., 2018a,b). We could not exclude that discrepancies between our findings and those reported by others are attributed to differences in sample sizes and patients’ clinical features, as well as different methodological (e.g., Surface-Based Analysis vs. Voxel-Based Morphometry) and statistical approach and whether the whole cerebellum or focal cerebellar regions were addressed.

We also found that severe motor disability and high rate of disease progression were slightly related to CV reduction of the sensory-motor cerebellar lobules. This is consistent with the evidence of gradually progressive cerebellar gray matter degeneration throughout disease progression in ALS (Bede and Hardiman, 2017). A recent study showed reduced gray matter volume (lobule IV and V) in ALS patients with no cognitive defect and posterior (VIII) cerebellar involvement in ALS patients with cognitive impairment (Christidi et al., 2018a). This is in line with our correlation analyses, confirming that specific patterns of subregional involvement are differently associated with cognitive and motor impairment in ALS (Tan et al., 2014).

Unexpectedly, our investigation of the cerebellar subregions did not revealed atrophy clusters specific for C9+ALS patients, which is consistent with recent findings (Floeter et al., 2016; Schönecker et al., 2018) but not with others (Westeneng et al., 2016; Agosta et al., 2017). Since cerebellar involvement in C9orf72 carriers have been found among FTD-ALS spectrum (Bede et al., 2013a; Irwin et al., 2013; Floeter et al., 2016), it is conceivable that it may reflect a signature of ALS dementia other than a signature of the C9orf72 hexanucleotide repeat (Bede et al., 2013a). Further studies deserve larger C9+ALS samples stratified on the basis of the degree of cognitive impairment to address this issue.

The strength of the presented study was the tentative to systematically explore the cognitive contributions of cerebellar atrophy in selected cohorts of patients with ALS. Our data show that cerebellar degeneration is not specific for non-demented patients with ALS and cognitive syndromes or C9orf72 mutations. However, the relatively small number of mutation carriers and the lack of ALS-FTD patients included limit the strength for firm conclusion. Indeed, we could not exclude that overall disease progression both in terms of progressive physical disability and progressive cognitive impairment might be related to cerebellar involvement, in line with its topographic organization.

## Data Availability

The datasets generated for this study are available on request to the corresponding author.

## Ethics Statement

The review board of the Fondazione IRCCS Istituto Neurologico Carlo Besta of Milan approved the study. Each subject was enrolled after giving written informed consent.

## Author Contributions

MC, EDB, AN, CP, CG, and SF collected the data. AN, CP, GD, MB, and SF acquired and the analyzed neuroimaging data. MC, SF, and SC designed the study. MC and LP performed the statistical analyses. MC, EDB, SC, MB, GL, and SF gave important intellectual content in data interpretation. MC, EDB, LP, and AN wrote a draft of the manuscript. MC, SC, GL, and SF revised the final version of the manuscript for intellectual content. MC, EDB, AN, CP, GD, LP, CG, VP, SC, MB, GL, and SF read and approved the final version.

## Conflict of Interest Statement

The authors declare that the research was conducted in the absence of any commercial or financial relationships that could be construed as a potential conflict of interest.
